# James Leo Gibbons (MD (Newcastle), FRCP, FRCPsych): Formerly Professor of Psychiatry and Honorary Consultant Psychiatrist, University of Southampton (1970–1986)

**DOI:** 10.1192/pb.bp.115.052928

**Published:** 2016-02

**Authors:** Gerald Russell

**Figure:**
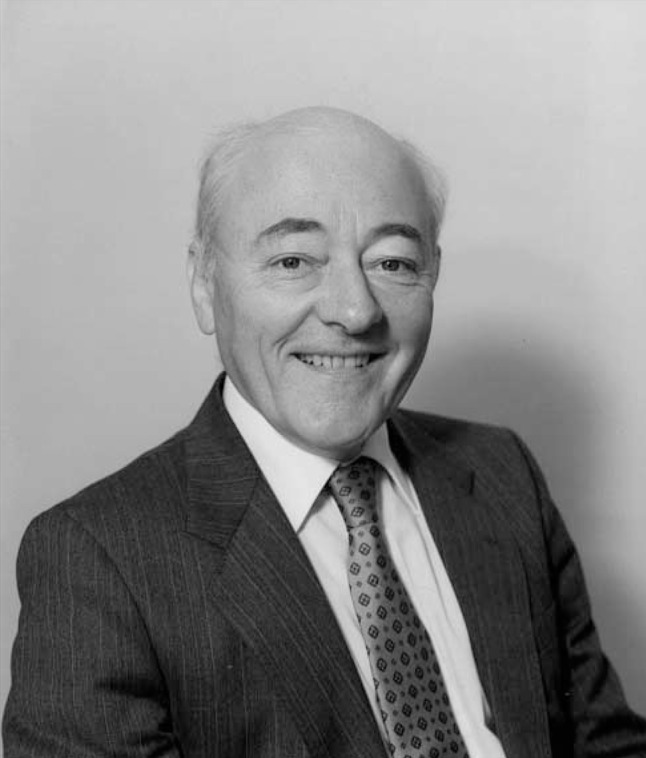


James Gibbons, who died recently at the age of 90, was responsible for the new finding that plasma cortisol levels are elevated in patients with depressive illness. This remarkable discovery came early in his career, while working in the metabolic unit at the Maudsley Hospital. The result was the landmark article which appeared in 1962^1^ and established the relationship between cortisol and depression. The impact of this article was profound and it soon became a citation classic, with 161 citations by other scientists, including 17 since 2010. The importance of this article is confirmed because the citations came from workers who were the pioneers in the field of psychoneuroendocrinology.^2^ Furthermore, it demonstrated the state-related nature of plasma cortisol elevations in patients with depression through an interaction with the rated severity of the clinical features.

In a supplementary study Gibbons used another index of adrenocortical function. This was based on a radiocarbon dilution method after administering a small dose of 4-C14 labelled cortisol. The cortisol secretion rates were estimated one week after admission and again after recovery or improvement from depression. A highly significant lowering was found on the repeat estimation. This second study confirmed the original finding, namely that patients with depression as a group showed increased adrenocortical activity. These findings led directly to the dexamethasone suppression test for melancholia.

Later on in his career, as professor of psychiatry at the University of Southampton, Gibbons changed the direction of his research, undertaking a survey of psychiatric services based on the district general hospital.^3^ Collaborating with his wife Jane, he followed up a large group of patients with schizophrenia using rating scales to supplement the clinical appraisals. At the end of one year the findings were, on the whole, rewarding. Only a small minority of the patients were still living in a mental hospital, half were living in private households with a supporter (spouse or parent), and the remainder were in a variety of sheltered accommodation. But almost half the patients were classified as psychotic on the Personal State Examination (PSE) Catego program. Disturbed behaviour and restricted social performance were frequent, and there was evidence of some hardship in most of the households.^3^ Regrettably, as a result of a cut in funding, some of the research had to be abandoned and the psychiatric register closed.

James was born in Felling (Co. Durham) on 8 June 1925, the son of a headmaster. He attended Ushaw College, a Catholic school, where he concentrated on the classics. His decision to follow a medical career was taken rather late and he needed to catch up on the sciences. After graduation and house appointments he served as medical officer in the Royal Air Force. While in junior posts he published articles on demyelinating diseases. He was appointed to the Maudsley Hospital in 1955. He soon became first assistant to Professor Aubrey Lewis and based in the metabolic unit which had special facilities for the clinical bioscience research which suited him very well. He was appointed senior lecturer at the Institute of Psychiatry and honorary consultant to the Maudsley Hospital in 1961. From 1963 to 1970 he worked as Reader in the Department of Psychological Medicine and honorary consultant psychiatrist, University of Newcastle. The remainder of his career was spent as professor of psychiatry at the University of Southampton.

Other distinctions were the award of travelling fellowships. In 1962 he obtained the William Waldorf Astor Fellowship enabling him to spend 4 months in the Department of Neuroendocrinology, Walter Reed Army Institute of Research, Washington DC. In 1964 he was awarded the Commonwealth Fellowship to visit medical schools in the USA and study the use of elective time in the medical curriculum.

After leaving the Maudsley, his clinical commitments grew at the cost of his time for research, especially while he was working in Southampton. He and his team had become responsible for the psychiatric service to a deprived area of the city. He accepted this as he believed that professors of psychiatry should be full clinicians as well as academics.

In personality, James tended to be serious minded and reserved. Strangely, these qualities suited him well in the role of an informal teacher. His listeners were always astounded by his erudition. He would share his knowledge generously whether this was over cups of coffee or in the laboratory. Invariably considerate in his personal relations, his kindness to his patients knew no bounds. Although he seldom showed extremes of emotion, there were exceptions, as when he openly wept while hosting his department's farewell to a seriously ill colleague. Although most of his colleagues knew James to be staunchly Catholic in his religious beliefs, mutual reticence led to avoiding this as a subject of conversation.

James took a slightly early retirement on health grounds in 1986. He then divided his time between Saxmundham and London. He loved good food and wine, and good restaurants. He visited Venice annually as long as he could. He enjoyed musical concerts and opera.

James's first marriage was to Joan Farrall, who died in 1971. In 1974 he married Jane Bunch, lecturer in social work at the University of Southampton. James died peacefully at home in Saxmundham on 17 June 2015. He is survived by Jane and Timothy, son of his first marriage, now working in Cambodia.

## References

[1] Gibbons JL McHugh PR Plasma cortisol in depressive illness. Psychiatr Res 1962; 1: 162-71. 10.1016/0022-3956(62)90006-7 13947658

[2] Coppen A The biochemistry of affective disorders. Br J Psychiatry 1967; 113: 1237-64. 4169954 10.1192/bjp.113.504.1237

[3] Gibbons JS Horn SH Powell JM Gibbons JL Schizophrenic patients and their families. A survey of a psychiatric service based on a DGH Unit. Br J Psychiatry 1984; 144: 70-7. 6692078 10.1192/bjp.144.1.70

